# Microbial succession on decomposing root litter in a drought-prone Scots pine forest

**DOI:** 10.1038/s41396-019-0436-6

**Published:** 2019-05-23

**Authors:** Claude Herzog, Martin Hartmann, Beat Frey, Beat Stierli, Cornelia Rumpel, Nina Buchmann, Ivano Brunner

**Affiliations:** 1 Swiss Federal Research Institute WSL, CH-8903 Birmensdorf, Switzerland; 2 ETH Zürich, CH-8092 Zürich, Switzerland; 3 Centre Nationale de Recherche Scientifique (CNRS), Institute of Ecology and Environment (IEES), Thiverval-Grignon, 78850 France

**Keywords:** Microbial ecology, Biogeochemistry, Forest ecology

## Abstract

Decomposition is a major flux of the carbon cycle in forest soils and understanding the involved processes is a key for budgeting carbon turnover. Decomposition is constrained by the presence of biological agents such as microorganisms and the underlying environmental conditions such as water availability. A metabarcoding approach of ribosomal markers was chosen to study the succession of bacterial and fungal decomposers on root litter. Litterbags containing pine roots were buried in a pine forest for two years and sequentially sampled. Decomposition and the associated communities were surveyed under ambient dry and long-term irrigation conditions. Early decomposition stages were characterized by the presence of fast-cycling microorganisms such as Bacteroidetes and Helotiales, which were then replaced by more specialized bacteria and litter-associated or parasitic groups such as Acidobacteria, white rots, and Pleosporales. This succession was likely driven by a decrease of easily degradable carbohydrates and a relative increase in persistent compounds such as lignin. We hypothesize that functional redundancy among the resident microbial taxa caused similar root decomposition rates in control and irrigated forest soils. These findings have important implications for drought-prone Alpine forests as frequent drought events reduce litter fall, but not litter decomposition, potentially resulting in lower carbon stocks.

## Introduction

Forest soils have sequestered approximately 1.1 ± 0.8 petagrams of carbon per year over the past decades [1]. Future predictions for terrestrial carbon uptake remain uncertain and will be strongly affected by more extreme climatic events [2]. Temperate forests, in particular, will face increasing frequencies and longer lasting periods of summer drought [3], which may result in increased tree mortality [4, 5] and establishment of more drought-adapted key tree species [6]. Forest soil carbon pools are mainly driven by primary production, litter decomposition, and stabilization, but it remains unclear whether there will be an increase or decrease in soil carbon with altered climatic conditions [7]. While primary production can be well monitored and modeled (e.g., SILVA [8], ANAFORE [9]), modeling carbon stocks in soils is more difficult and, among other factors, hampered by the lack of data on root decomposition [10, 11]. In the past decade, various attempts have been made to quantify and understand the complex process of litter decomposition [12, 13]. Nevertheless, direct in vivo manipulation studies are rare, and it remains unclear how climatic factors affect litter decomposition.

While leaf and needle production as well as foliar litter decomposition can be successfully and routinely assessed, root production and decomposition still lack suitable and widely accepted measurement methods [12, 14]. Measuring root decomposition is important because there is increasing evidence that root carbon may contribute more to forest soil carbon pools than aboveground biomass [15–18]. Root composition and characteristics may also be key factors influencing litter decomposition in soils [13, 19]. Compared to foliar litter, roots experience greater physical and chemical protection from decomposition [15]. Greater chemical recalcitrance of roots compared with that of leaf litter, related to a higher lignin and lower nutrient concentration, may impede their microbial degradability [12, 20]. This chemical protection by a high amount of persistent lignin has become a major area of research because of the prospect of being able to identify one of the key components controlling SOM stabilization [21, 22]. Nonetheless, its importance is contested [23].

Silver and Miya [24] concluded from their meta-analyses that the lignin to nitrogen ratio (lignin: N) is one of the main drivers of litter decomposition, and this finding has been confirmed by many others in later studies [22, 25]. While nitrogen often acts as a driver of decomposition, lignin, because of its highly branched structure of interlinked aromatic rings (monophenols) [26], hinders decomposition. The measurement of lignin monophenols evolved as a state-of-the-art biomarker to determine the quality, quantity, and decomposition state of litter [27, 28]. Some researchers have followed the decomposition state at different depths along the soil profile [25, 27, 29], whereas others have combined measurement of microbial biomarkers with cupric oxide lignin characterization [28]. Unfortunately, only a limited number of long-term studies have been performed, particularly regarding lignin, that could help to trace the plant-derived compounds to their final preserved state [25].

In addition to the effects of litter composition, climatic variables such as temperature and water availability strongly affect decomposition in soils [13, 19]; hence increased water availability does not directly affect the tree litter composition [30]. If litter quality is not directly affected by water availability, the positive relationship between water availability and decomposition could be related to biological factors. Studies investigating drought effects on microbial communities show a decrease in microbial biomass and activity with long-lasting droughts [31]. In addition, severe droughts can decrease enzyme production in the rhizosphere [32]. In the soil of a Scots pine forest in a drought-prone environment, a long-term irrigation treatment suspended summer droughts, resulting in a stimulation of microbial activity [33]. Further, the authors showed a clear shift from predominantly oligotrophic microbial communities in the dry control plots to more copiotrophic communities in the irrigated plots. This shift could be explained by a higher turnover rate of carbon in the irrigated plots, in which case enhanced degradation of easily degradable components of roots with irrigation would be expected. Overall, irrigation in a drought-prone forest should result in increased microbial degradation of root litter.

Little is known about how the microbial community affects the degradation of root litter in forest soils. Reed and Martiny [34] reviewed several common gardens and reciprocal transplant studies and concluded that microbial community composition affected decomposition in only some cases. In another review, McGuire and Treseder [10] summarized the relevance of shifts in microbial community structure for the decomposition process; they reported that the presence of certain functional groups, rather than microbial biomass, seems decisive for decomposition. Several studies have been conducted to assess litter decomposition in agricultural systems [35, 36], but often with a limited taxonomic resolution [28, 37] or considering short timescales [38]. Two studies using next-generation sequencing techniques [39, 40] detected a vertical succession of fungal functional groups within the soil profile. Recent studies have investigated the decomposition of leaf litter over a longer period, along with analyzing bacterial and fungal community composition [41, 42]. With the application of modern sequencing techniques, limitations have shifted from the relatively low taxonomic resolution of the acquired results to the restrictions of useful and updated comparisons, which means that taxa might remain uncharacterized and sequences unannotated [43, 44].

Specialized lignin degraders such as the white-rot fungi seem to be the only organisms capable of complete mineralization of the lignin molecule [26]. If and how bacteria play a role in lignin degradation is not entirely resolved [45, 46]. Baldrian [47] pointed out that studies addressing the combined role of bacteria and fungi during decomposition are still rare, with the exception of a few recently published studies [41, 42]. Whereas reports on facilitative (e.g., lichen, fungal highways) and antagonistic interactions (e.g., parasitism, competition) between bacteria and fungi are numerous, it is less clear how positive and negative interactions between bacteria and fungi affect degradation of carbon sources in soils [48, 49]. Whereas theoretical models about cheaters and producers or opportunists and miners have been developed [50, 51], it is certain that all these interactions are highly regulated by environmental conditions such as nutrient availability, by costs of enzyme production, and by substrate accessibility.

Succession of microbes on naturally occurring persistent macromolecules has become a focus of many microbiological studies under controlled laboratory conditions, e.g., rapid micro-scale succession on chitin particles in seawater [52]. Microbial succession studies investigating the degradation of leaf litter have been performed recently in various forest types [41, 42, 53–55]; however, the degradation of root litter was not considered. Controlled laboratory studies have been conducted to decipher degradation of various wood types [56]. Nonetheless, there is still more work to do, as Schimel and Schaeffer [57] stated: “The largest uncertainty about the role of community composition probably exists for dead roots.”

To address this lack of studies with roots, we studied the decomposition of roots in a forest soil, traced the fate of lignin in these roots, and identified bacterial and fungal succession throughout the course of root decomposition. These investigations were carried out in a water-limited Scots pine forest in Switzerland, where half of the experimental plots are irrigated with the intention of relieving the trees from summer drought stress. We combined chemical assays with a metabarcoding approach of bacterial and fungal ribosomal markers to address the following questions: (1) Do chemical and biochemical traits of decomposing roots change over a 2-year period, and which traits are affected by the irrigation treatment? (2) Do bacterial and fungal communities in soils and roots differ from each other? (3) Can we observe successional patterns of bacterial and fungal taxa within the decomposing roots over a period of two years, and how are the patterns affected by irrigation? (4) Do lignin-decomposing fungi enter the decomposing roots only at a later stage of decomposition or are they present from the beginning, and how does irrigation affect the succession patterns of these fungi?

## Material and methods

### Site description

The root decomposition study has been performed in the Pfyn forest (Pfynwald). This forest is situated in the Rhone Valley of Switzerland (46°18′N, 7°37′E, 615 m a.s.l.) in a drought-prone Scots pine forest (*Pinus sylvestris*).

Mean annual values (1981–2016) of the nearby climatic station at Sion were 10.3 °C and 594 mm precipitation [58]. The soil has an 8–12 cm thick topsoil comprising of a 3–6 cm thick organic layer (Oe layer) and a 2–6 cm thick humic mineral soil layer (A layer, with organic matter accumulation). Below the topsoil, the subsoil has a high percentage of skeletal material (20–50% or above) with rocks originating from the bed load of the Ill river. The pH of the topsoil has a mean of 5.4 and that of the subsoil 7.5 [59]. Due to the long-term irrigation treatment, the Oe layer (partially decomposed litter) in the irrigated plots converted to an Oa layer (fully decomposed litter, humus). However, the thickness of the layers did not change strongly due to the irrigation treatment.

In this mature pine forest, eight plots (25 × 40 m each) were installed, and four of these plots have been irrigated during the summer months (April–October) since 2003 [30]. A reduction of severe and long-lasting summer drought periods had been achieved by approximately doubling precipitation from about 500 to 1000 mm per year, resulting in an increase of the mean crown cover from 57% to 71% within a decade [33]. The irrigation treatment resulted in a significant increase in the mean volumetric soil water content (SWC) from 27.8% to 34.3% [30].

### Root decomposition experiment

Roots for the decomposition study were extracted with a spade from each of the Pfynwald plots in autumn 2013 and transported in plastic bags to the lab. There, the roots in each sample were washed on a sieve under running water and pine fine roots (*Ø* < 2 mm) were picked out. Roots with a greater diameter were not considered for this study. Subsequently, the roots were air dried and stored at room temperature. In February 2014, litterbags with a mesh size of 1 mm and a size of 10 × 10 cm were filled with 1 g of air-dried root material. The mesh size of 1 mm allows the mesofauna (e.g., mites, springtails) to enter, but not the macrofauna (e.g., earthworms). The litterbags were made of a robust nylon mesh (Sefar Petex^®^, Sefar AG, Heiden, Switzerland). At the end of March 2014, before the start of the irrigation, each root-filled litterbag was buried in the same plot from which roots were originally excavated. In order to destructively sample the litterbags at five different time points, five litterbags per time-point and plot were tied together with a nylon cord and buried horizontally between the O and A layers at 5 cm depth measured from the surface (O layer). Before installing the litterbags, the litter (Oi layer, undecomposed litter) was removed. The sampling time points for the litterbags containing roots were 0, 3, 6, 12, 18, and 24 months. In total, 240 litterbags with roots were collected. Roots from the first time point (T0) were not buried in the soils. Litterbags of later time points were excavated carefully and stored in plastic bags and cool boxes during transportation to the laboratory. In the laboratory, the roots were carefully cleaned with a fine brush to remove adhesive soil, frozen in liquid nitrogen, and freeze-dried. Subsequently, roots from the same plot and time point were pooled, dry weights were measured, and samples were then stored at −20 °C until further processing. From the remaining root litter mass, the decomposition rate constant *k* was calculated according to the reaction rate formula:

$$\begin{array}{*{20}{c}} {\ln (M_t /M_0 ) = - k\times t } \end{array}$$

where *M*_0_ is the initial mass and *M*_t_ is the remaining mass after decomposition time *t*.

At the time of litterbag collection, soil samples were also collected next to the litterbags. Three soil samples, after the litter layer (Oi layer) was removed, were taken with a hand spade from the topsoil (2–10 cm, consisting of Oe/Oa layer and A horizon) and pooled per plot. Soil samples were transported in plastic bags and cool boxes to the laboratory. The soil samples were sieved through a 2 mm sieve, and about 1–2 g soil aliquots were frozen in liquid N_2_, lyophilized, and stored at −80 °C until DNA extraction. About 5–10 g soil was dried at 105 °C for soil water content calculations, and the remaining soil was dried at 60 °C for final storage.

### Soil water content and temperature measurements

During the whole study period, SWC was recorded with EC-5 sensors (Decagon Devices, Pullman, WA, USA) and soil temperature with RT-1 sensors (Decagon Devices, Pullman, WA, USA) at 5 cm depth near the litterbags within each plot. Data were stored with EM50 digital data loggers (Decagon Devices, Pullman, WA, USA).

### Root chemical analyses

Carbon (C), nitrogen (N), and the stable C and N isotopes of the milled root material were analyzed with an elemental analyzer-continuous flow isotope ratio mass spectrometer (Euro-EA, Hekatech GmbH, Germany, interfaced with a Delta-V Advanced IRMS, Thermo GmbH, Germany). The Vienna Pee Dee Belemnite international standard was used for the calculation of the ratio between ^13^C and ^12^C (δ^13^C), and the isotope ratio ^15^N to ^14^N of the air was used as a standard for the calculation of the δ^15^N. Both δ^13^C and δ^15^N were expressed in per mill (‰).

Lignin monomers from the milled root material were extracted with the cupric oxide oxidation method introduced by Hedges and Ertel [60] and modified by Kögel-Knabner and Bochter [61]. Briefly, the root material was oxidized by cupric oxide in a pressure oven (175 °C, 2.5 h). A recovery standard was added (ethyl vanillin) and the solution was acidified with HCl (pH 2). After precipitation of the humic acids, lignin monomers were extracted using solid-phase extraction with a C18 absorbent column (SUPELCO, Sigma-Aldrich, Buchs, Switzerland) with one drop per second at 20 °C. The monophenols were eluted with ethylacetate and dried under N_2_ and re-dissolved in pyridine. An internal standard (phenyl acetic acid) was added to the extracted monophenols. N,O-bis(trimethylsilyl)trifluoroacetamide (BSTFA) and trimethylchlorosilane (TCMS; 99:1) were used as derivatization agents (Sigma-Aldrich, Buchs, Switzerland). The monophenols were characterized on a Thermo Scientific™ TRACE™ 1300 Gas Chromatograph coupled to a Thermo Scientific™ ISQ™ Single Quadrupole Mass Spectrometer (GC-MS) and quantified on a Thermo Scientific™ Flame Ionization Detector (FID for TRACE™ 1300 GC Series). For quantification, external standards of the monophenols present in lignin were measured alongside the samples (Supplementary Table S1). The ratios of acid to aldehyde vanillyl phenols (Ad/Al_V_) and acid to aldehyde ratio of syringyl units (Ad/Al_S_) were calculated.

### DNA extraction from roots and soils

For DNA extraction, either lyophilized root material, which was hand milled with a mortar under liquid nitrogen, or frozen soil material was used. DNA was extracted using the MoBio Power Soil DNA isolation kit (MoBio Laboratories, Carlsbad, CA, USA). For the bead beating procedure according to Frey et al. [62], either 0.5 g soil or 0.1 g root material was used. DNA concentration was determined using PicoGreen (Molecular Probes, Eugene, OR, USA). Ten nanograms of DNA was used to perform PCR amplification of the ribosomal small-subunit RNA gene (region V3–V4) and the internal transcribed spacer (region ITS2). PCR amplification was done in triplicate and pooled as previously described [33, 63–65]. Pooled DNA samples were sent to the Génome Québec Innovation Center at McGill University (Montréal, Canada) for barcoding using the Fluidigm Access Array technology (Fluidigm) and paired-end sequencing on the Illumina MiSeq v3 platform (Illumina Inc., San Diego, CA, USA).

### Sequence quality control, determination of exact sequence variants and taxonomic assignments

Sequences were processed using a customized pipeline largely based on VSEARCH v2.8 [66]. Paired end reads were merged using the fastq_mergepairs algorithm [67] implemented in VSEARCH. Merged reads deriving from PhiX spiked into the sequencing run were removed by running Bowtie2 [68] against the PhiX genome. PCR primers were trimmed using Cutadapt [69] allowing for one mismatch. Trimmed reads were quality filtered using the fastq_filter function [67] implemented in VSEARCH allowing for a maximum expected error of one. Sequences were dereplicated using the derep_fulllength function in VSEARCH and singletons were removed. Exact sequence variants (ESVs) were retrieved from the dereplicated dataset using the UNOISE algorithm [70] implemented in VSEARCH with an alpha of 2 and minsize of 4. Potentially chimeric ESV sequences were identified and removed using the UCHIME2 algorithm [71] implemented in VSEARCH as the uchime3_denovo function. Remaining ESV sequences were tested for the presence of ribosomal signatures using Metaxa2 [72] and ITSx [73] for the 16S rRNA gene and ITS2 sequences, respectively, and unsupported sequences were discarded. The final ESV table was obtained by mapping the quality filtered reads of each sample against the verified ESV sequences using the usearch_global algorithm implemented in VSEARCH. Taxonomic classification of each verified ESV sequence was performed by running the SINTAX algorithm [74] implemented in VSEARCH against the SILVA v.128 database [75] for the 16S rRNA gene sequences and against the UNITE v.7.2 database [76] for the ITS2 sequences using a bootstrap cutoff of 0.8. Non-fungal ITS2 sequences were identified and removed from the ESV table by classifying the ESV sequences against a customized ITS2 database featuring all eukaryotic ITS2 sequences deposited in the NCBI nucleotide database [77]. Finally, 16S rRNA gene ESV sequences assigned to organelle structures (chloroplasts, mitochondria) were removed from the ESV table. Raw sequences were deposited in the European Nucleotide Archive under the accession number PRJEB21241.

### Quantitative PCR of bacterial and fungal ribosomal markers

Relative abundances of the bacterial 16S rRNA gene and fungal ITS copies were determined by quantitative real-time PCR (qPCR) according to Frossard et al. [78] with an ABI7500 fast real-time PCR system (Applied Biosystems, Foster City, CA, USA). The same primers (without barcodes) and cycling conditions as used for the sequencing approach were used for the 16S rRNA gene and ITS2 targets. The initial DNA denaturation was at 95 °C for 15 min. Each of the following 40 amplification cycles involved a denaturation step at 95 °C for 30 s, primer annealing at 60 °C for 45 s, and an extension phase for 45 s at 72 °C. The final cycle included a denaturation step at 95 °C for 15 s, primer annealing at 60 °C for 1 min, followed by denaturation at 95 °C for 15 s. For qPCR analyses the root-DNA was diluted to 0.33 ng µL^−1^ (0.02–0.4 ng µL^−1^ for the time point 0 due to low DNA-concentrations) and used in a 15 µL master mix containing 7.5 µL QuantiTect SYBR Green PCR master mix (Qiagen, Hilden, Germany), 2 × 0.15 µL primer (100 µM), 0.5 µL RNase-free water, 0.1 µL BSA (30 mg mL^−1^), and 6.6 µl template. Standard curves per target region (correlations ≥ 0.997) were generated using tenfold serial dilutions (10^−2^–10^−8^ copies) of plasmids derived from cloned targets [79]. Data were converted to represent an average copy number of targets per gram of root dry weight.

### Statistics

All statistical tests were performed with *R* [80]. A *P*-value < 0.05 was considered significant in all statistical tests. Alpha-diversity was estimated by calculating the Shannon-index implemented in the ‘diversity’ function of the vegan package [81] of rarefied data by smallest site maximum (number of sequences) using ‘rarefy’ implemented in vegan. Beta-diversity between substrates was calculated by Bray–Curtis dissimilarity using ‘vegdist’ implemented vegan package. For assessing similarity in microbial community structure, principle coordinate analysis (PCoA) [82] was performed. Constrained analysis of principal coordinates (CAP) was performed and the explanatory variables (SWC, weight remaining, Ad/Al_V_, Ad/Al_S_, δ^13^C, δ^15^N, and C/N) were plotted against the CAP-coordinates for bacteria and fungi. The arrows and statistical legend tables were calculated using the function ‘envfit’ implemented in vegan. Similarities between groups were assessed by the mantel function [83] implemented in vegan with 10^5^ permutations. Effects of factors on the relative abundance overall differences using multivariate PERMANOVA and of individual taxa within the microbial community were assessed by univariate permutational ANOVA based on Euclidian distances using the ‘adonis’ function in vegan with 10^5^ permutations. To correct for multiple testing, the ‘p-adjust’ function (method: ‘fdr’, [84]) was used, resulting in a corrected *P*-value, with a corrected *P*-value < 0.05 considered significant. A heatmap was calculated from Hellinger and *Z*-score transformed relative abundance data using basic *R* [80].

## Results

Long-term irrigation strongly increased the soil volumetric water content (VWC) compared to ambient conditions (irrigated: 0.12 (±0.02 SE) m^3^ m^−3^, control: 0.08 (±0.01 SE) m^3^ m^−3^, Supplementary Fig. S1). In contrast, soil temperature was not affected by irrigation (irrigated: 10.3 (±0.10) °C, control: 10.2 (±0.21) °C; Supplementary Fig. S1). In the control treatments the VWC was in the first year of the decomposition study several times close to 0 m^3^ m^−3^, in June, September, and October, and in the second year in July, September, and October, whereas in the irrigation treatment, the VWC was usually above 0.10 m^3^ m^−3^. In the winter months, however, the VWC was in both treatments more or less identical. Overall, the summer (July–September) of the second year of decomposition was much drier in terms of precipitation (126 mm) compared with the first year (208 mm), as recorded at the nearby weather station in Sion [58].

After two years of decomposition, 55% (±2%) and 58% (±2%) of the root litter mass remained in the control and irrigated plots, respectively, with the treatments not being significantly different from each other (Table 1). A clear decrease by approximately one fourth of the mass was detected after three months and decomposition rates (*k*) were not altered significantly by the irrigation treatment (control: 0.30 (±0.02 SE) year^−1^; irrigated: 0.28 (±0.02 SE) year^−1^), even though root litter from irrigated plots showed a significantly higher root water content during the irrigation period (early and late summer) compared to the non-irrigated period. In contrast, during the non-irrigated period, root litter water content was mostly higher in the control than in the irrigated plots.

**Table 1:** Means of root chemical properties over time of decomposition with the standard error in parentheses (*n* = 4)

Timepoint	0 Month		3 Months		6 Months		12 Months		18 Months		24 Months		ANOVA		
Treatment	Control	Irrigated	Control	Irrigated	Control	Irrigated	Control	Irrigated	Control	Irrigated	Control	Irrigated	Treatment	Timepoint	Interaction
Mass remaining [g]	1	1	0.77 (0.02)	0.78 (0.01)	0.74 (0.02)	0.73 (0.01)	0.70 (0.00)	0.70 (0.01)	0.65 (0.06)	0.65 (0.02)	0.55 (0.02)	0.58 (0.02)	0.894	**6.0E−16**	0.785
Root water content [g]	0	0	0.93 (0.16)	1.90 (0.05)	0.73 (0.12)	1.70 (0.22)	0.56 (0.05)	0.41 (0.04)	0.30 (0.03)	0.36 (0.01)	0.45 (0.06)	0.30 (0.02)	0.377	**1.2E−15**	**3.3E−05**
Carbon content [%]	44.9 (0.46)	45.2 (0.94)	46.2 (0.48)	43.3 (2.05)	46.1 (0.63)	44.6 (0.48)	46.2 (1.17)	45.7 (0.72)	45.3 (1.06)	44.1 (0.97)	45.6 (0.71)	44.2 (1.93)	**2.8E−04**	0.130	0.092
δ^13^C [‰]	−25.9 (0.19)	**−**26.6 (0.31)	−26.0 (0.18)	−26.6 (0.32)	−26.1 (0.15)	−26.6 (0.41)	−26.0 (0.17)	−26.7 (0.36)	−25.9 (0.39)	−26.6 (0.43)	−26.1 (0.24)	−26.4 (0.36)	**2.3E−09**	0.959	0.829
Nitrogen content [%]	0.78 (0.03)	0.65 (0.05)	0.82 (0.08)	0.78 (0.06)	0.91 (0.08)	0.76 (0.03)	0.90 (0.06)	0.79 (0.04)	0.89 (0.09)	0.79 (0.04)	0.86 (0.11)	0.77 (0.03)	**1.9E−06**	**0.003**	0.564
δ^15^N [‰]	−9.31 (0.70)	−9.18 (0.44)	−10.12 (0.45)	−9.41 (0.67)	−10.28 (0.19)	−9.45 (1.07)	−10.05 (0.64)	−8.97 (0.74)	−9.09 (1.20)	−8.95 (1.23)	−9.41 (0.63)	−8.75 (0.98)	**0.009**	0.053	0.662
Carbon/Nitrogen	57.7 (2.73)	69.5 (6.26)	56.7 (4.84)	55.5 (5.83)	50.7 (4.73)	59.1 (2.67)	51.6 (2.99)	58.1 (3.78)	51.1 (5.50)	55.9 (3.02)	53.4 (5.86)	57.8 (4.12)	**9.5E−05**	**0.001**	0.133
Lignin Monomers															
V.S.C. [mg/g]	15.8 (1.66)	12.5 (0.81)	15.3 (2.22)	17.0 (1.08)	15.7 (1.74)	13.7 (2.05)	24.6 (4.28)	23.6 (3.14)	24.2 (6.89)	16.8 (3.72)	19.4 (3.57)	15.1 (1.85)	0.119	**0.028**	0.461
Ad/Al_V_	0.32 (0.05)	0.26 (0.02)	0.29 (0.03)	0.24 (0.02)	1.20 (0.60)	0.98 (0.09)	0.46 (0.28)	0.27 (0.05)	0.86 (0.18)	0.61 (0.14)	1.14 (0.65)	0.57 (0.14)	**3.0E−05**	**3.7E−13**	0.432
Ad/Al_S_	0.52 (0.56)	0.91 (0.57)	1.53 (0.76)	1.13 (1.23)	0.70 (0.73)	1.74 (0.24)	0.55 (0.58)	2.42 (1.44)	0.13 (0.14)	0.23 (0.06)	0.44 (0.39)	1.30 (0.36)	**0.001**	**0.001**	0.088
Lignin/Nitrogen	20.2 (3.59)	19.2 (2.79)	19.1 (7.11)	21.8 (3.64)	17.3 (3.86)	18.0 (4.85)	27.4 (9.82)	30.1 (8.47)	28.0 (17.93)	21.2 (8.94)	22.7 (8.94)	19.6 (4.10)	0.808	0.178	0.894

Lignin monomers in milligram per gram root litter dry weight. Overall lignin given as the sum over the vanillyl, syringyl, and cinnamyl monomers (V.S.C.), the ratio of acid to aldehyde vanillyl monomers (Ad/Al_V_), and the ratio of acid to aldehyde syringyl monomers (Ad/Al_S_). ANOVA *P*-values < 0.05 are given in bold

### Shifts in litter chemical properties

Shifts of root litter chemical properties over time are summarized in Table 1. The total carbon (C) in the root litter was significantly reduced by irrigation. However, root C was not significantly affected by time, although showing a slight increase after 6 and 12 months, then decreasing back to the starting amount after 18 months. Over the whole study period, root litter showed a significantly lower nitrogen (N) concentration in the irrigated plots compared to the control plots. Total N in roots increased significantly over the study period in both control and irrigated plots. Therefore, the C to N ratio (C/N) in roots decreased significantly with progressing decomposition under the irrigation treatment. δ^13^C in roots decreased significantly with irrigation, from about −26.0 to −26.5‰, but it remained unaffected over the treatment period. In contrast, δ^15^N increased significantly under irrigation, from about −9.7‰ to −9.1‰. δ^15^N remained unaffected over the whole treatment period.

The extracted lignin monophenols relative to root dry weight are presented in Table 1. The overall sum of monophenols (vanillyl, syringyl, and cinnamyl, V.S.C.) changed significantly during progressing decomposition. V.S.C. remained stable during the first 6 months then showed a significant increase, with a peak at 12 months, then decreased again for the time points 18 and 24 months. With progressing decomposition, significantly increasing ratios of acid to aldehyde vanillyl phenols (Ad/Al_V_) were found. The irrigation treatment showed a decreasing Ad/Al_V_ ratio. The acid to aldehyde ratio of syringyl units (Ad/Al_S_) decreased as well significantly over time. With progressing decomposition, the lignin to N ratio did not change significantly.

### Microbial response to substrate and irrigation treatment

The gene copy number of bacteria (16S rRNA gene) and fungi (ITS2 rrn) of the roots increased within three months around 16-fold for bacteria and 17-fold for fungi, respectively, after root substrate colonization in soil (Supplementary Fig. S2). After that, bacterial as well as fungal abundances remained stable until the end of the experiment at 24 months. Overall, bacterial and fungal abundances changed significantly over time, whereas the irrigation treatment did not influence microbial abundance significantly (Supplementary Table S2). The bacteria to fungi ratio remained constant during the 2-year period with values between 3.6 and 3.8 and was not affected by the irrigation treatment. Only the irrigated roots before they were buried had a ratio of 5.0, but the ratio dropped to 3.7 after the roots were put back into the soil (Supplementary Fig. S2).

**Fig. 1:**
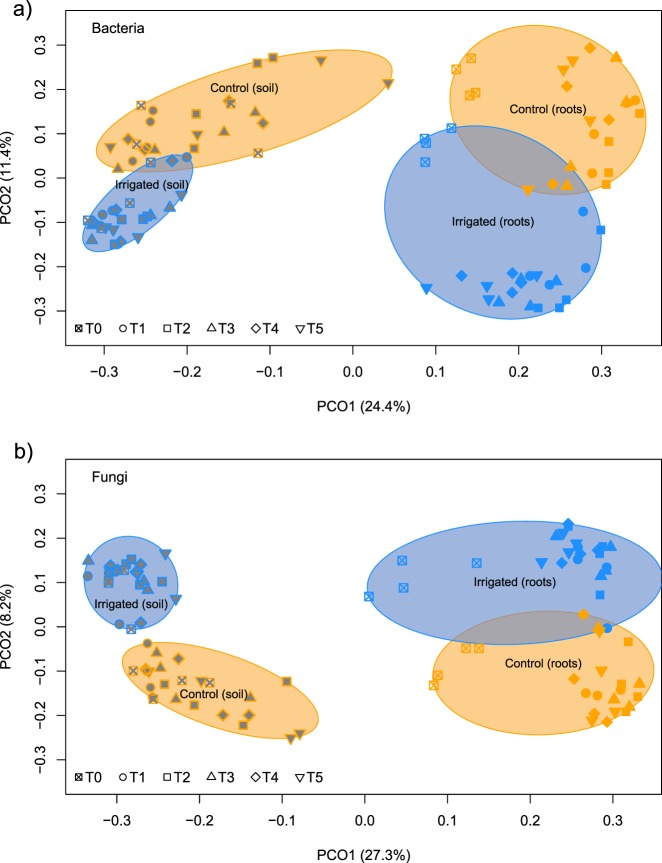
Principal coordinate ordination (PCO) showing differences in bacterial (**a**) and fungal (**b**) community structure on decomposing roots (colored filling) and the surrounding soil (gray filling) over a 2-year period in irrigated (blue) and control (orange) forest plots. Different symbols indicate different stages of decomposition (time). The ellipses encloses all points in the group for factor combination of treatment (control, irrigated) and source (soil, roots). The variance explained by each PCO axis is given in parentheses

Microbial diversity differed strongly between sources (root versus soil), with bacteria and fungi showing similar response patterns (Table 2, Fig. 1). Soil communities showed a higher alpha diversity but lower beta diversity compared to root litter communities. Similarities were detected in overall species composition by comparing the PCoA scores underlying dissimilarity matrices using Mantel test (Table 2), hence, explaining around 26% of the variance, only. The applied irrigation treatment had a minor but still significant effect on the microbial community composition (Table 2, Fig. 1). Source had the greatest effect on the bacterial and fungal community composition (Adonis *F*-value: 37.0, *P*-value: <0.001), while the irrigation treatment was significant but less decisive (Adonis *F*-value: 11.1, *P*-value: <0.001).

**Table 2:** Metrics for alpha and beta diversity of bacterial and fungal communities, i.e., the number of ESVs and mean ESVs per sample with the standard error in brackets, Shannon diversity index with standard error, and beta-diversity (mean distance to centroid) for control and irrigated plots

		Control				Irrigated				Mantel (*R*)	Mantel (*P*-value)
		Richness	ESV/sample	Alpha-diversity	Beta-diversity	Richness	ESV/sample	Alpha-diversity	Beta-diversity	Root litter	Root litter
Bacteria	Soil	28260	9267 (192)	8.68 (0.03)	0.66 (0.02)	28000	9568 (198)	8.75 (0.02)	0.65 (0.01)	0.264	**<0.001**
	Root litter	23466	5859 (381)	8.00 (0.10)	0.71 (0.04)	25599	6868 (490)	8.11 (0.18)	0.69 (0.06)	1	0
Fungi	Soil	2664	837 (27)	5.39 (0.06)	0.68 (0.02)	2520	832 (21)	5.60 (0.04)	0.66 (0.01)	0.260	**0.001**
	Root litter	2084	584 (19)	5.03 (0.05)	0.70 (0.02)	2042	589 (31)	4.94(0.08)	0.69 (0.03)	1	0

Mantel test *R* and *P*-value, *P*-value < 0.05 are given in bold

### Microbial succession on root litter

**Fig. 2:**
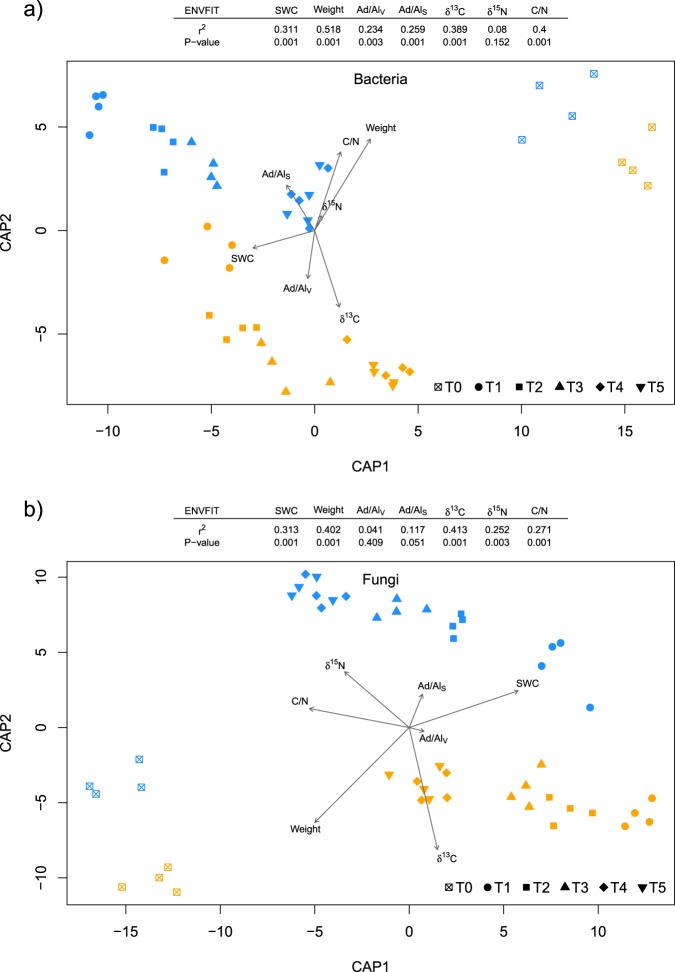
Constrained analysis of principal coordinates (CAP) of **a** bacterial and **b** fungal communities on decomposing roots maximizing discrimination between irrigation treatment and the different stages of decomposition. Different symbols indicate different stages of decomposition (time), whereas the color-coding refers to the communities in irrigated (blue) and control (orange) plots. Gray vectors show the correlation between the measured environmental variables (soil water content, remaining weight, Ad/Al_V_, Ad/Al_S_, δ^13^C, δ^15^N, C/N) and the ordinations scores, with the length of the vector corresponding to the correlation coefficient (*r*). Statistical significance of the squared correlation coefficient (*r*^2^) and the level of significance (*P*-value) of the environmental variable fit are provided in a legend table

A clear gradual succession of the fungal and bacterial communities on the root litter was detected at the ESV level (Fig. 2). Factor time did significantly explain the shifts in community structure for bacteria (Adonis *F*-value: 3.47, *P*-value: <0.001) and fungi (Adonis *F*-value: 2.70, *P*-value: <0.001) on the decomposing roots, hence to a lower degree than the irrigation treatment (bacteria: Adonis *F*-value: 10.7, *P*-value: <0.001; fungi: Adonis *F*-value: 7.62, *P*-value: <0.001). The Mantel test of the underlying dissimilarity matrices revealed a significant similar shift between bacterial and fungal community structures (compare Fig. 1a, b, Mantel *r* = 0.89, and Mantel *P*-value = <0.001).

Constrained analysis by time and irrigation treatment using CAP revealed a clear gradual community shift across the different times points for both bacteria and fungi, and shifts correlated with changes in root chemical properties (Fig. 2). Strongest correlations of the bacterial community were detected with the remaining weight of the root litter, with the carbon-to-nitrogen ratio (C/N) and with the δ^13^C. In addition, the lignin degradation index of Ad/Al_V_ and Ad/Al_S_ significantly correlated with the bacterial community distribution, but not with the fungal. Similarly, the remaining weight of the root litter and δ^13^C showed high correlation with the fungal canonical coordinates. Hence, δ^15^N showed a strong significant correlation with fungal community distribution exclusively. Soil water content correlated strongly with both fungal and bacterial scores, respectively.

### Response of bacterial phyla to treatment and shifts over time

**Fig. 3:**
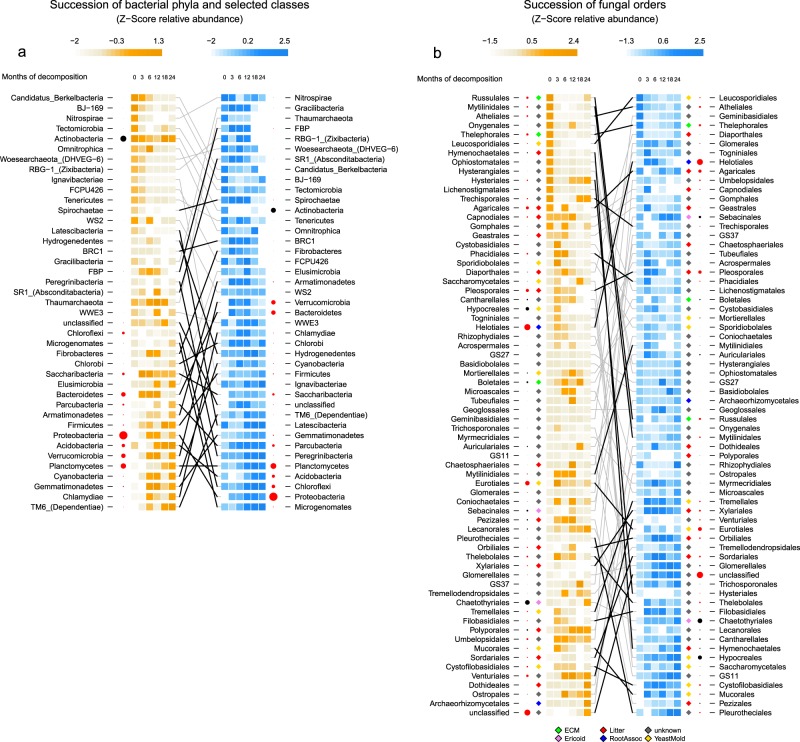
Heat map of *Z*-scores centered over time in control (orange) and irrigated plots (blue) for **a** bacterial phyla and **b** fungal orders. The size of the circles corresponds to the relative abundance (square-root) of each taxon. Taxa that changed significantly over time are highlighted in red. Gray lines connect the same taxa, whereas black lines highlight those that changed significantly under irrigation. Colored diamonds represent functional groups of fungi at the order level according to Lindahl et al. [101] and Kyaschenko et al. [135], i.e., yeast and molds (yellow), litter-associated (red), ectomycorrhizae (green), ericoid mycorrhizae (purple), root associated (blue), and unknown (gray)

Proteobacteria was the predominant bacterial phylum as in relative abundance of assigned ESVs. The factors treatment and mainly time point significantly affected their abundance on the root litter (Fig. 3a). In contrast, the relatively abundant phylum Actinobacteria did not change significantly across the course of the experiment, hence, in the irrigated plots they were mainly present in the unburied roots. Planctomycetes, Chloroflexi, Acidobacteria, Bacteroidetes, and Verrucomicrobia were dominant taxa on the decomposing roots, all significantly affected in their abundance by both time point and treatment. Bacteroidetes and Verrucomicrobia were most abundant during the first year (T1–T3), whereas Planctomycetes, Chloroflexi, and Acidobacteria dominated in the second year (T4–T5).

Parcubacteria (former OD-1) showed a significant shift over time and increased prevalence in irrigated plots.

### Response of fungal orders to treatment and shifts over time

Few orders which were already present on the roots before burying (T0) remained on the roots after burying, exceptions were the orders Agaricales and Russulales, which were present at T0 and at later time points. The dominant orders in the first year on the decomposing roots were Helotiales, Eurotiales, and Pleosporales; in addition for irrigated roots Xylariales, which were marginally present in the control (Fig. 3b). In the second year, the fungal orders dominating the roots differ quite strongly between the control and irrigated plots with the exception of the order Chaetothyriales, which is highly abundant in both treatments (Fig. 3b). In the control plots, mainly the orders of Venturiales, Umbelopsidales, and Polyporales were present in the last time points, apart from the most abundant group of unclassified fungal ESVs. In the irrigated plots, the dominant fungal orders in the second year were Hypocreales, Hymenochaetales, Agaricales, and Sebacinales. In general, the first year was dominated by fast growing Ascomycota and were then replaced by more specialized litter-associated or mycorrhizal Basidiomycota (Supplementary Fig. S3).

## Discussion

Root decomposition is one of the main drivers of the biogeochemical cycle and carbon storage in temperate forest soils [7]. Therefore, assessing and understanding this process is of major importance for accurate carbon budgeting. Under changing environmental conditions, obtained either by experimental manipulation or by using natural gradients caused by global climate change, the rate of root decomposition and therefore carbon sequestration can vary strongly [2]. We expected to find an increase in root decomposition with increasing water availability at the naturally dry Pfynwald forest ecosystem, as very dry conditions can lead to a reduction or even impairment in decomposition processes [85]. In this study, however, no significant difference was observed between irrigated and control plots in the root mass remaining after two years of decomposition. A decrease in root mass of nearly 20% was detected after three months of decomposition in control as well as in irrigated plots. This finding is in agreement with previous decomposition studies [86] and might be higher as compared to natural conditions, since buried root lack physical soil protection at the earliest time point.

Mortality of Scots pine [6] is mainly driven by direct effects of drought or indirectly by increased sensitivity to further adverse elements such as pathogen attack. Hence, limiting conditions for Scots pine, do not have to coincide with limiting conditions for microbes. Our results revealed stable microbial decomposition rates over two years, irrespective of irrigation treatment. Nonetheless, water availability and soil moisture are factors known to be key drivers of decomposition [13, 19]. Hartmann et al. [33] showed a significant increase in soil respiration due to irrigation in Pfynwald. Furthermore, Hagedorn (unpublished data) observed a significant increase in earthworm abundance in the irrigated plots. Although we focused on microbial decomposition in this study and excluded earthworms with the selected mesh size, it is important to note that these soil engineers also play a key role in decomposition [87]. Water limitation is not as relevant to microorganisms as to larger soil fauna, although the mass loss associated with soil fauna can drastically reduce under dry conditions [88]. Furthermore, we can assume that the limited water availability to plants is not an equally limiting factor to microorganisms. In drying soil, soil water retention increases and water becomes inaccessible to plant roots, whereas bacteria and fungi might still have access to water in small soil pores and thus might be less limited by the increasing soil water surface tension. Moreover, numerous soil bacteria are able to form spores, cysts, or biofilms or can reduce their metabolism to endure unfavorable conditions [89–91], and groups such as fungi or Actinobacteria generally show a high tolerance to desiccation [90, 92]. Thus, we conclude that the dry conditions in Pfynwald did not limit microbial decomposition.

### Shifts of elements and lignin monophenols in root litter

The element analysis of our root litter (Table 1) showed results similar to those from roots measured in 2013 within the same irrigation experiment [30]. Both N concentration and the C/N ratio were affected by irrigation. Roots in the irrigated plots have a lower N concentration and an increased C/N ratio compared to those in the dry plots.

This reduced N concentration in the irrigated roots might indicate plant N limitation, since Scots pine had abundant water supply and carbon assimilation therefore was not limited. During root decomposition, an increase in N concentration was observed, most likely an indirect effect of increasing colonization by bacteria and fungi, which contain N in their cell walls [93]. The δ^13^C values of tree rings increased under dry conditions in the control plots, most likely due to lower stomatal conductance and thus less discrimination against ^13^CO_2_ during photosynthesis [94]. This difference between the control and the irrigation treated roots remained stable during the entire course of root litter decomposition. Irrigation led to increased root δ^15^N compared to values in the control plots, suggesting that the N mineralization rate increased in irrigated plots [95].

Litter quality, and in particular lignin concentration, is an important factor driving decomposition [24, 86]. The sum of the measured lignin monophenols V.S.C. after cupric oxide extraction were within the range of 10–30 mg g^−1^ DW, which is in agreement with the values for *Pinus* species reported in the literature [96]. Our results showed that irrigation did not significantly influence the lignin fingerprints. A clear temporal pattern over the decomposition period was detected, with a distinct twofold increase in lignin concentration after 12 months and a subsequent decrease after the second year. A similar pattern, with an initial increase and a long-term decrease in lignin, was detected in a previous study in which concentrations of organic compounds were traced during five years of decomposition [86]. Duboc et al. [28] confirmed these observations with their findings of slower lignin decomposition rates compared to that of bulk litter C but no difference over longer time scales (two years). This result implies that lignin is selectively preserved during the initial decomposition phase compared to other litter compounds. With respect to irrigation, we observed significantly lower degrees of lignin oxidation, expressed with the Ad/Al_v_ ratio [27], during the 2-year experiment in irrigated plots compared with the dry control plots. These lignin degradation indices of Ad/Alv and Ad/Als correlate stronger with the bacterial than with the fungal community composition (Fig. 2). Since this indicator also reflects the acid to aldehyde ratio, we can hypothesize a higher sensitivity to pH by bacteria compared to fungi [97].

### Microbial communities in soil and root litter

In theory, the dominance of soils by copiotrophic microorganisms leads to higher carbon turnover rates [98]. Indeed, in Pfynwald, this effect was detected in soils of the irrigated plots, with a higher turnover of carbon resulting in a relative increase in copiotrophic microorganisms such as Proteobacteria and Mucoromycota [33]. However, this difference in soil microbial community composition did not lead to an alteration in root litter degradation. Interestingly, the soil microbial community composition did differ significantly from the root litter community in our study, hence, recruitment of microorganisms from the surrounding soil by the decomposing roots is likely [99] and clearly supported by our data with the strong microbial shift after root burying (Figs. 1 and 2, T0–T1). In addition to the strong influence of the habitat (soil versus root), the irrigation treatment lead to a strong shift in both bacterial and fungal community. Less decisive, but still of significance, was the litter quality change over time selecting for microbes specialized for a given degree of decomposition [100]. Such community changes due to the change of the source has been observed as well in boreal forests, with a shift from saprotrophic fungi on fresh litter in top soil to ectomycorrhizal fungi on humus in deeper soil layers [39, 101].

### Shifts of root litter microbial communities

In the present study, the fungal community-shift over time, due to progressing litter decay, was similar to that observed in the vertical chronosequence in Sweden [39]. Early successional groups, mainly dominated by fast growing Ascomycota (e.g., Helotiales, Pleosporales, Eurotiales), are rather known to consume easily degradable plant components. Xylariales mainly present on the root litter of the irrigated plots has been identified as an important order of cellulose degradation in the topsoil of a *Picea abies* forest [102], and furthermore revealed morphological properties of the wood rot similar to those produced by white-rot fungi [103]. While the first study year was dominated by similar fungal orders between the two treatments, the second year revealed different fungal orders on the roots. The root litter of the control plots in the second year was mainly colonized by the orders Venturiales, Umbelopsidales, and Polyporales. Members of the Venturiales order are well known for their saprotrophic or parasitic lifestyle [104] and are therefore potential candidates for degradation of more persistent litter components. Not much is known about the recently defined order of Umbelopsidales, detected on living roots [105] and isolated as endophytes [106]. With our detection on decomposing litter, a saprotrophic lifestyle should be taken into consideration. Polyporales are typical degraders of woody debris either degrading lignin as white-rot fungi [107], or more likely cellulose and hemi-cellulose as brown-rot fungi after losing their capacity for lignin degradation [108, 109]. The irrigated litterbags were dominated by the orders Hypocreales, Russulales, Sebacinales, Agaricales, and Hymenochaetales. Hypocreales have been detected as a major pest causing root rot on *Pinus sylvestris* [110] and isolated from decaying wood thereby revealing saprotrophic characteristics [111]. In our samples, the saprotrophic genus *Trichoderma sp*. was more abundant than the root rot causing *Fusarium sp*. A similar dominant abundance was revealed by mycorrhizal orders (e.g., Sebacinales, Russulales, Chaetothyriales), these findings are supported by a Swedish study detecting these orders in humus like litter [39]. Sebacinales are a functionally diverse order [112] and are not only able to form mycorrhizal associations but also follow saprotrophic lifestyles [113, 114]. Recently, some authors have proposed that mycorrhizal fungi are able to oxidize organic matter not necessarily to access carbon but rather to scavenge nitrogen [115–117]. In the irrigated plots, the litter associated Agaricales have been detected during both years of decomposition, while in the control plots they were mainly present in the unburied roots (T0). Hymenochaetales members present in the irrigated litterbags are known lignin degraders [108, 109] and dominate woody debris decomposition in forest alongside the order of Polyporales [118].

In a similar manner, bacteria did reveal a clear successional trend. Hartmann et al. [33] investigated dry and irrigated soils of the Pfynwald forest and argued that many members of the phylum Proteobacteria occurring in the irrigated plots exhibited copiotrophic characteristics [98]. In our study, many bacterial phyla showed a similar occurrence pattern between control and irrigation treatment. In the unburied roots (T0), Actinobacteria was the dominant phylum, but only remained highly abundant in the control plots, likely from the high abundance in the surrounding soil [33]. In the irrigated plots, however, their abundance strongly decreased after burying the roots. Actinobacteria are well renowned as degraders of plant material in soil, in a similar way as saprotrophic fungi, and therefore strong competitors on root litter [40]. The first year (T1–T3) was characterized by high relative abundance of Bacteroidetes and Verrucomicrobia in the irrigated and control plots. Bacteroidetes are known as litter-associated bacteria [119] that show a broad substrate range. In case of the order Sphingobacteriales, they mainly degrade cellulose [120] or mycelium (Chitinophagaceae) [119]. Verrucomicrobia have been detected as saprotrophic degraders after wild oat root addition in an agricultural system [121]. The last successional step in the second year (T4–T5) showed mainly an increase in Acidobacteria and Planctomycetes in both treatments and Chloroflexi mainly in the irrigated plots. Interestingly, the phylum Firmicutes, an often detected major player amongst the bacterial root degraders [40, 122], was only detected at a marginal abundance in our study. Not much is known about the ecological importance of Parcubacteria, as they have escaped cultivation so far. Members of this group have been characterized by strongly reduced genomes and a preferred occurrence in largely anoxic environments [64, 123, 124]. It has been hypothesized that the streamlined genome, carrying a limited metabolic capacity, might be an indication of a symbiotic lifestyle, either mutualistic or parasitic [124]. Recently, Castelle et al. [125] found in addition to the anaerobic fermentative metabolism also a respiratory capacity; however, the authors predict a “similar obligate dependence on other organisms or the microbial community.” The importance of bacteria regarding litter decay in soils might have been underestimated [126]. In our study, we detected a strong succession of bacteria over time that could not be explained by physicochemical variation in the soil. Thus, the strong bacterial succession might be the result of versatile functional niche occupation during progressing root degradation.

### Lignin degrading fungi and bacteria

Major wood-decaying fungal orders such as Polyporales, Hymenochaetales, Russulales, and Agaricales [107] were present during late successional stages in control and irrigated plots in Pfynwald. Similarly, an increased detection of white-rot fungi with progressing decomposition was recently detected in a survey on tree logs [127]. However, these orders have not previously been observed in situ on buried roots. A recent publication revealed that most species in the order of Polyporales lost their capability for complete lignin degradation [109] and rather perform a form of brown-rot degradation where cellulose and hemicellulose are degraded preferentially. In our study, the data indicate differential selection for fungal wood degraders by the irrigation treatment, since Hymenochaetales were prevalent on the roots in the irrigated plots and Polyporales in the control plots. Ottosson et al. [118] discussed the issue of competition between several wood-degrading fungi in a very intriguing manner and even though there is little published evidence of direct competition, the basic principle of overlapping niches has to select for a single most competitive wood degrader. Competition is very likely not exclusively occurring among fungal groups. The highly significant and negative correlation between the abundance of Actinobacteria and Xylariales in our data might be an indication for resource competition between fungi and bacteria. A decrease in actinobacterial abundance on litter has been observed with presence of arbuscular mycorrhizal fungi [128], and competition between Actinobacteria and fungal cellulose degraders has been hypothesized [129].

For bacteria, ligninolytic capabilities have mainly been reported for Actinobacteria [26]. In this study, Thermoleophilia were the second most abundant actinobacterial class. Within the most abundant phylum Proteobacteria, only few genera have so far revealed ligninolytic capacities [130]. The most abundant genera within the phylum Proteobacteria were *Sphingomonas sp*. (4.4%), *Burkholderia-Paraburkholderia sp*. (3.2%), and *Pseudomonas sp*. (2.5%), all of which have been identified as ligninolytic, or capable of degrading aromatic structures [130]. Two etherases produced by *Sphingomonas paucimobilis* after cultivation on a lignin-related biphenyl medium have been identified [131], and *Burkholderia-Paraburkholderia sp*. which were very abundant on the control roots (>30% of Betaproteobacteria), have been proven to produce ligninolytic enzymes in culture [132]. Moreover, Valášková et al. [133] postulated a co-occurrence of Betaproteobacteria with white-rot fungi profiting from the fungal exuded degradation enzymes. *Pseudomonas sp*. and *Burkholderia-Paraburkholderia sp*. were found on and around fungal structures, most likely profiting from the degradation product of the fungal enzymes or directly from fungal exudates [129], or, in the case of *Pseudomonas sp*., directly degrading fungal mycelia [119]. Therefore, it remains uncertain if under natural conditions bacteria degrade lignin, or simply profit from fungal lignin degradation. Most studies dealing with lignin degradation focus on single strains under laboratory conditions, whereas proof of lignin degradation under field conditions or under natural competition is still lacking. There is a need for a better understanding of degradation and involved carbon fluxes from plant derived carbon sources through the microbial food web.

## Conclusions

In conclusion, our results revealed a successional shift, from fast growing to specialized bacteria and fungi, on the decomposing roots independent of soil water conditions. Nevertheless, root-associated microbial communities differed between irrigated and ambient control plots. However, we did not observe a shift in decomposition rates, mediated by these distinct microbial communities, from the dry (control) to the moist environment (irrigated). In microbial studies such as ours, environmental constraints often need to reach very extreme levels, beyond our experimental setup with long-term drought and drought release by irrigation, to exert limiting conditions for microorganisms, exceeding viable conditions for many higher organisms (e.g., vascular plants). Moreover, in recent years, the hypothesis of functional redundancy among microbial communities–suggesting that different species occupying the same niche contribute to the same process at a similar rate–has gained increasing attention [36, 134]. Such functionally redundant species might be dispersed across the taxonomic hierarchy, especially in the case of bacteria. Our data suggest that such a functional redundancy might occur among the microbial communities in terms of root decomposition at the study site Pfynwald.

## Supplementary information

Supplementary materials

## References

[1] Pan Y, Birdsey RA, Fang J, Houghton R, Kauppi PE, Kurz WA (2011). A large and persistent carbon sink in the world’s forests. Science.

[2] Reichstein M, Bahn M, Ciais P, Frank D, Mahecha MD, Seneviratne SI (2013). Climate extremes and the carbon cycle. Nature.

[3] Millar CI, Stephenson NL (2015). Temperate forest health in an era of emerging megadisturbance. Science.

[4] Anderegg WRL, Kane JM, Anderegg LDL (2013). Consequences of widespread tree mortality triggered by drought and temperature stress. Nat Clim Chang.

[5] Hartmann H, Ziegler W, Kolle O, Trumbore S (2013). Thirst beats hunger-declining hydration during drought prevents carbon starvation in Norway spruce saplings. New Phytol.

[6] Rigling A, Bigler C, Eilmann B, Feldmeyer-Christe E, Gimmi U, Ginzler C (2013). Driving factors of a vegetation shift from Scots pine to pubescent oak in dry Alpine forests. Glob Chang Biol.

[7] De Deyn GB, Cornelissen JH, Bardgett RD (2008). Plant functional traits and soil carbon sequestration in contrasting biomes. Ecol Lett.

[8] Pretzsch H (2001). Modellierung des Waldwachstums.

[9] Deckmyn G, Verbeeck H, De Beeck MO, Vansteenkiste D, Steppe K, Ceulemans R (2008). ANAFORE: a stand-scale process-based forest model that includes wood tissue development and labile carbon storage in trees. Ecol Model.

[10] McGuire KL, Treseder KK (2010). Microbial communities and their relevance for ecosystem models: decomposition as a case study. Soil Biol Biochem.

[11] Didion M, Frey B, Rogiers N, Thürig E (2014). Validating tree litter decomposition in the Yasso07 carbon model. Ecol Model.

[12] Berg B, McClaugherty C, Berg B, McClaugherty C (2014). Decomposition of fine root and woody litter. Plant litter.

[13] Solly EF, Schöning I, Boch S, Kandeler E, Marhan S, Michalzik B (2014). Factors controlling decomposition rates of fine root litter in temperate forests and grasslands. Plant Soil.

[14] Goebel M, Hobbie SE, Bulaj B, Zadworny M, Archibald DD, Oleksyn J (2011). Decomposition of the finest root branching orders: linking belowground dynamics to fine-root function and structure. Ecol Monogr.

[15] Rasse DP, Rumpel C, Dignac M-F (2005). Is soil carbon mostly root carbon? Mechanisms for a specific stabilisation. Plant Soil.

[16] Fan P, Guo D (2010). Slow decomposition of lower order roots: a key mechanism of root carbon and nutrient retention in the soil. Oecologia.

[17] Sanaullah M, Chabbi A, Leifeld J, Bardoux G, Billou D, Rumpel C (2011). Decomposition and stabilization of root litter in top-and subsoil horizons: what is the difference?. Plant Soil.

[18] Amin BAZ, Chabbert B, Moorhead D, Bertrand I (2014). Impact of fine litter chemistry on lignocellulolytic enzyme efficiency during decomposition of maize leaf and root in soil. Biogeochemistry.

[19] Prescott CE (2010). Litter decomposition: what controls it and how can we alter it to sequester more carbon in forest soils?. Biogeochemistry.

[20] Kögel-Knabner I (2002). The macromolecular organic composition of plant and microbial residues as inputs to soil organic matter. Soil Biol Biochem.

[21] Hofmann A, Heim A, Christensen BT, Miltner A, Gehre M, Schmidt M (2009). Lignin dynamics in two 13C labelled arable soils during 18 years. Eur J Soil Sci.

[22] Walela C, Daniel H, Wilson B, Lockwood P, Cowie A, Harden S (2014). The initial lignin: nitrogen ratio of litter from above and below ground sources strongly and negatively influenced decay rates of slowly decomposing litter carbon pools. Soil Biol Biochem.

[23] Cotrufo MF, Wallenstein MD, Boot CM, Denef K, Paul E (2013). The microbial efficiency-matrix stabilization (MEMS) framework integrates plant litter decomposition with soil organic matter stabilization: do labile plant inputs form stable soil organic matter?. Glob Chang Biol.

[24] Silver WL, Miya RK (2001). Global patterns in root decomposition: comparisons of climate and litter quality effects. Oecologia.

[25] Heim A, Frey B (2004). Early stage litter decomposition rates for Swiss forests. Biogeochemistry.

[26] Abdel-Hamid AM, Solbiati JO, Cann IKO, Sariaslani S, Gadd GM (2013). Insights into lignin degradation and its potential industrial applications. Advances in applied microbiology.

[27] Thevenot M, Dignac M-F, Rumpel C (2010). Fate of lignins in soils: a review. Soil Biol Biochem.

[28] Duboc O, Dignac M-F, Djukic I, Zehetner F, Gerzabek MH, Rumpel C (2014). Lignin decomposition along an Alpine elevation gradient in relation to physicochemical and soil microbial parameters. Glob Chang Biol.

[29] Rumpel C, Kögel-Knabner I, Bruhn F (2002). Vertical distribution, age, and chemical composition of organic carbon in two forest soils of different pedogenesis. Org Geochem.

[30] Herzog C, Steffen J, Pannatier EG, Hajdas I, Brunner I (2014). Nine years of irrigation cause vegetation and fine root shifts in a water-limited pine forest. PloS One.

[31] Schimel JP, Gulledge JM, Clein-Curley JS, Lindstrom JE, Braddock JF (1999). Moisture effects on microbial activity and community structure in decomposing birch litter in the Alaskan taiga. Soil Biol Biochem.

[32] Sanaullah M, Blagodatskaya E, Chabbi A, Rumpel C, Kuzyakov Y (2011). Drought effects on microbial biomass and enzyme activities in the rhizosphere of grasses depend on plant community composition. Appl Soil Ecol.

[33] Hartmann M, Brunner I, Hagedorn F, Bardgett RD, Stierli B, Herzog C (2017). A decade of irrigation transforms the soil microbiome of a semi-arid pine forest. Mol Ecol.

[34] Reed HE, Martiny JBH (2007). Testing the functional significance of microbial composition in natural communities. FEMS Microbiol Ecol.

[35] Dilly O, Bloem J, Vos A, Munch JC (2004). Bacterial diversity in agricultural soils during litter decomposition. Appl Environ Microbiol.

[36] Allison SD, Lu Y, Weihe C, Goulden ML, Martiny AC, Treseder KK (2013). Microbial abundance and composition influence litter decomposition response to environmental change. Ecology..

[37] Treseder KK, Bent E, Borneman J, McGuire KL (2014). Shifts in fungal communities during decomposition of boreal forest litter. Fungal Ecol.

[38] Purahong W, Arnstadt T, Kahl T, Bauhus J, Kellner H, Hofrichter M (2016). Are correlations between deadwood fungal community structure, wood physico-chemical properties and lignin-modifying enzymes stable across different geographical regions?. Fungal Ecol.

[39] Clemmensen KE, Bahr A, Ovaskainen O, Dahlberg A, Ekblad A, Wallander H (2013). Roots and associated fungi drive long-term carbon sequestration in boreal forest. Science.

[40] Sanaullah M, Chabbi A, Maron P-A, Baumann K, Tardy V, Blagodatskaya E (2016). How do microbial communities in top-and subsoil respond to root litter addition under field conditions?. Soil Biol Biochem.

[41] Purahong W, Kapturska D, Pecyna MJ, Jariyavidyanont K, Kaunzner J, Juncheed K (2015). Effects of forest management practices in temperate beech forests on bacterial and fungal communities involved in leaf litter degradation. Microb Ecol.

[42] Purahong W, Wubet T, Lentendu G, Schloter M, Pecyna MJ, Kapturska D (2016). Life in leaf litter: novel insights into community dynamics of bacteria and fungi during litter decomposition. Mol Ecol.

[43] McDonald D, Price MN, Goodrich J, Nawrocki EP, DeSantis TZ, Probst A (2012). An improved Greengenes taxonomy with explicit ranks for ecological and evolutionary analyses of bacteria and archaea. ISME J.

[44] Gilbert JA, Jansson JK, Knight R (2014). The Earth microbiome project: successes and aspirations. BMC Biol.

[45] Brown ME, Chang MC (2014). Exploring bacterial lignin degradation. Curr Opin Chem Biol.

[46] De Gonzalo G, Colpa DI, Habib MHM, Fraaije MW (2016). Bacterial enzymes involved in lignin degradation. J Biotechnol.

[47] Baldrian P (2017). Forest microbiome: diversity, complexity and dynamics. FEMS Microbiol Rev.

[48] Meidute S, Demoling F, Bååth E (2008). Antagonistic and synergistic effects of fungal and bacterial growth in soil after adding different carbon and nitrogen sources. Soil Biol Biochem.

[49] Haq Irshad Ul, Zhang Miaozhi, Yang Pu, van Elsas Jan Dirk (2014). The Interactions of Bacteria with Fungi in Soil. Advances in Applied Microbiology.

[50] Allison SD (2005). Cheaters, diffusion and nutrients constrain decomposition by microbial enzymes in spatially structured environments. Ecol Lett.

[51] Moorhead DL, Sinsabaugh RL (2006). A theoretical model of litter decay and microbial interaction. Ecol Monogr.

[52] Datta MS, Sliwerska E, Gore J, Polz MF, Cordero OX (2016). Microbial interactions lead to rapid micro-scale successions on model marine particles. Nat Commun.

[53] Voříšková J, Baldrian P (2013). Fungal community on decomposing leaf litter undergoes rapid successional changes. ISME J.

[54] Haňáčková Z, Koukol O, Štursová M, Kolařík M, Baldrian P (2015). Fungal succession in the needle litter of a montane *Picea abies* forest investigated through strain isolation and molecular fingerprinting. Fungal Ecol.

[55] Tláskal V, Voříšková J, Baldrian P (2016). Bacterial succession on decomposing leaf litter exhibits a specific occurrence pattern of cellulolytic taxa and potential decomposers of fungal mycelia. FEMS Microbiol Ecol.

[56] Prewitt L, Kang Y, Kakumanu ML, Williams M (2014). Fungal and bacterial community succession differs for three wood types during decay in a forest soil. Microb Ecol.

[57] Schimel JP, Schaeffer SM (2012). Microbial control over carbon cycling in soil. Front Microbiol.

[58] MeteoSwiss. MeteoSwiss-Monthly homogenized values. (2017). http://www.meteoschweiz.admin.ch/product/output/climate-data/homogenous-monthly-dataprocessing/data/homog_mo_SIO.txt.

[59] Brunner I, Pannatier EG, Frey B, Rigling A, Landolt W, Zimmermann S (2009). Morphological and physiological responses of Scots pine fine roots to water supply in a dry climatic region in Switzerland. Tree Physiol.

[60] Hedges JI, Ertel JR (1982). Characterization of lignin by gas capillary chromatography of cupric oxide oxidation products. Anal Chem.

[61] Kögel I, Bochter R (1985). Characterization of lignin in forest humus layers by high-performance liquid chromatography of cupric oxide oxidation products. Soil Biol Biochem.

[62] Frey B, Pesaro M, Rüdt A, Widmer F (2008). Dynamics of bacterial communities in bulk and poplar rhizosphere soil contaminated with heavy-metals. Environ Microbiol.

[63] Rime T, Hartmann M, Brunner I, Widmer F, Zeyer J, Frey B (2015). Vertical distribution of the soil microbiota along a successional gradient in a glacier forefield. Mol Ecol.

[64] Frey B, Rime T, Phillips M, Stierli B, Hajdas I, Widmer F (2016). Microbial diversity in European alpine permafrost and active layers. FEMS Microbiol Ecol.

[65] Frossard A, Hartmann M, Frey B (2017). Tolerance of the forest soil microbiome to increasing mercury concentrations. Soil Biol Biochem.

[66] Rognes T, Flouri T, Nichols B, Quince C, Mahé F (2016). VSEARCH: a versatile open source tool for metagenomics. Peer J.

[67] Edgar RC, Flyvbjerg H (2015). Error filtering, pair assembly and error correction for next-generation sequencing reads. Bioinformatics.

[68] Langmead B, Salzberg SL (2012). Fast gapped-read alignment with Bowtie 2. Nat Methods.

[69] Martin M (2011). Cutadapt removes adapter sequences from high-throughput sequencing reads. EMBnet J.

[70] Edgar RC. UNOISE2: improved error-correction for Illumina 16S and ITS amplicon sequencing. BioRxiv. 2016:081257.

[71] Edgar R. UCHIME2: improved chimera prediction for amplicon sequencing. BioRxiv. 2016:074252.

[72] Bengtsson-Palme J, Hartmann M, Eriksson KM, Pal C, Thorell K, Larsson DGJ (2015). METAXA2: improved identification and taxonomic classification of small and large subunit rRNA in metagenomic data. Mol Ecol Resour.

[73] Bengtsson-Palme J, Ryberg M, Hartmann M, Branco S, Wang Z, Godhe A (2013). Improved software detection and extraction of ITS1 and ITS2 from ribosomal ITS sequences of fungi and other eukaryotes for analysis of environmental sequencing data. Methods Ecol Evolut.

[74] Edgar R. SINTAX: a simple non-Bayesian taxonomy classifier for 16S and ITS sequences. BioRxiv. 2016:074161.

[75] Pruesse E, Quast C, Knittel K, Fuchs BM, Ludwig W, Peplies J (2007). SILVA: a comprehensive online resource for quality checked and aligned ribosomal RNA sequence data compatible with ARB. Nucleic Acids Res.

[76] Abarenkov K, Henrik Nilsson R, Larsson KH, Alexander IJ, Eberhardt U, Erland S (2010). The UNITE database for molecular identification of fungi–recent updates and future perspectives. New Phytologist.

[77] Benson DA, Clark K, Karsch-Mizrachi I, Lipman DJ, Ostell J, Sayers EW (2015). GenBank. Nucl Acids Res.

[78] Frossard A, Donhauser J, Mestrot A, Gygax S, Bååth E, Frey B (2018). Long-and short-term effects of mercury pollution on the soil microbiome. Soil Biol Biochem.

[79] Frey B, Niklaus PA, Kremer J, Lüscher P, Zimmermann S (2011). Heavy machinery traffic impacts methane emission, abundance of methanogens and community structure in oxic forest soils. Appl Environ Microbiol.

[80] R Core Team. R: a language and environment for statistical computing (version 3.3.2). (2014). http://www.R-project.org/.

[81] Oksanen J, Blanchet FG, Kindt R, Legendre P, Minchin PR, O’hara R et al. Package ‘vegan’. Community Ecology Package, Version. 2013;2.

[82] Gower JC (1966). Some distance properties of latent root and vector methods used in multivariate analysis. Biometrika.

[83] Legendre P, Legendre L. Numerical ecology. 3rd ed. Amsterdam: Elsevier; 2012.

[84] Benjamini Y, Hochberg Y (1995). Controlling the false discovery rate: a practical and powerful approach to multiple testing. J R Stat Soc Ser B.

[85] Sanaullah M, Rumpel C, Charrier X, Chabbi A (2012). How does drought stress influence the decomposition of plant litter with contrasting quality in a grassland ecosystem?. Plant Soil.

[86] Berg B, McClaugherty C, Berg B, McClaugherty C (2008). Decomposition, humus formation, carbon sequestration. Plant litter.

[87] Wardle D, Lavelle P, Cadisch G, Giller KE (1997). Linkages between soil biota, plant litter quality and decomposition. Driven by nature: plant litter quality and decomposition.

[88] Wachendorf C, Irmler U, Blume HP, Cadisch G, Giller KE (1997). Relationships between litter fauna and chemical changes of litter during decomposition under different moisture conditions. Driven by nature: plant litter quality and decomposition.

[89] Fierer N, Schimel J, Holden P (2003). Influence of drying–rewetting frequency on soil bacterial community structure. Microb Ecol.

[90] Barnard RL, Osborne CA, Firestone MK (2013). Responses of soil bacterial and fungal communities to extreme desiccation and rewetting. ISME J.

[91] Evans SE, Wallenstein MD (2014). Climate change alters ecological strategies of soil bacteria. Ecol Lett.

[92] De Vries FT, Liiri ME, Bjørnlund L, Bowker MA, Christensen S, Setälä HM (2012). Land use alters the resistance and resilience of soil food webs to drought. Nat Clim Chang.

[93] Wardle D (1992). A comparative assessment of factors which influence microbial biomass carbon and nitrogen levels in soil. Biolog Rev.

[94] Timofeeva G, Treydte K, Bugmann H, Rigling A, Schaub M, Siegwolf R (2017). Long-term effects of drought on tree-ring growth and carbon isotope variability in Scots pine in a dry environment. Tree Physiol.

[95] Templer PH, Arthur MA, Lovett GM, Weathers KC (2007). Plant and soil natural abundance δ15N: indicators of relative rates of nitrogen cycling in temperate forest ecosystems. Oecologia.

[96] Moingt M, Lucotte M, Paquet S (2016). Lignin biomarkers signatures of common plants and soils of Eastern Canada. Biogeochemistry.

[97] Rousk J, Bååth E, Brookes PC, Lauber CL, Lozupone C, Caporaso JG (2010). Soil bacterial and fungal communities across a pH gradient in an arable soil. ISME J.

[98] Fierer N, Bradford MA, Jackson RB (2007). Toward an ecological classification of soil bacteria. Ecology.

[99] Goldmann K, Schröter K, Pena R, Schöning I, Schrumpf M, Buscot F (2016). Divergent habitat filtering of root and soil fungal communities in temperate beech forests. Sci Rep.

[100] Cleveland CC, Reed SC, Keller AB, Nemergut DR, O’Neill SP, Ostertag R (2014). Litter quality versus soil microbial community controls over decomposition: a quantitative analysis. Oecologia.

[101] Lindahl BD, Ihrmark K, Boberg J, Trumbore SE, Högberg P, Stenlid J (2007). Spatial separation of litter decomposition and mycorrhizal nitrogen uptake in a boreal forest. New Phytol.

[102] Baldrian P, Kolařík M, Štursová M, Kopecký J, Valášková V, Větrovský T (2011). Active and total microbial communities in forest soil are largely different and highly stratified during decomposition. ISME J.

[103] Worrall James J., Anagnost Susan E., Zabel Robert A. (1997). Comparison of Wood Decay among Diverse Lignicolous Fungi. Mycologia.

[104] Zhang Y, Crous PW, Schoch CL, Bahkali AH, Guo LD, Hyde KD (2011). A molecular, morphological and ecological re-appraisal of venturiales―a new order of dothideomycetes. Fungal Divers.

[105] Yang H, Zhao X, Liu C, Bai L, Zhao M, Li L (2018). Diversity and characteristics of colonization of rootassociated fungi of *Vaccinium uliginosum*. Sci Rep.

[106] Terhonen E, Keriö S, Sun H, Asiegbu FO (2014). Endophytic fungi of Norway spruce roots in boreal pristine mire, drained peatland and mineral soil and their inhibitory effect on *Heterobasidion parviporum* in vitro. Fungal Ecol.

[107] Floudas D, Binder M, Riley R, Barry K, Blanchette RA, Henrissat B (2012). The paleozoic origin of enzymatic lignin decomposition reconstructed from 31 fungal genomes. Science.

[108] Krizsán K, Nagy LG, Grigoriev IV, Riley R, Hibbett DS, Bergmann PJ (2016). Genetic bases of fungal white rot wood decay predicted by phylogenomic analysis of correlated gene-phenotype evolution. Mol Biol Evol.

[109] Nagy LG, Riley R, Bergmann PJ, Krizsán K, Martin FM, Grigoriev IV (2016). Genetic bases of fungal white rot wood decay predicted by phylogenomic analysis of correlated gene-phenotype evolution. Mol Biol Evol.

[110] Kwaśna H, Bateman G (2009). Microbial communities in roots of *Pinus sylvestris* seedlings with damping-off symptoms in two forest nurseries as determined by ITS1/2 rDNA sequencing. For Pathol.

[111] Druzhinina I.S., Kubicek C.P. (2016). Familiar Stranger. Advances in Applied Microbiology.

[112] Vohník M, Pánek M, Fehrer J, Selosse M-A (2016). Experimental evidence of ericoid mycorrhizal potential within Serendipitaceae (Sebacinales). Mycorrhiza.

[113] Zuccaro A, Lahrmann U, Güldener U, Langen G, Pfiffi S, Biedenkopf D (2011). Endophytic life strategies decoded by genome and transcriptome analyses of the mutualistic root symbiont *Piriformospora indica*. PLoS Pathog.

[114] Weiß M, Waller F, Zuccaro A, Selosse MA (2016). Sebacinales–one thousand and one interactions with land plants. New Phytol.

[115] Lindahl BD, Tunlid A (2015). Ectomycorrhizal fungi–potential organic matter decomposers, yet not saprotrophs. New Phytol.

[116] Bödeker I, Lindahl BD, Olson Å, Clemmensen KE (2016). Mycorrhizal and saprotrophic fungal guilds compete for the same organic substrates but affect decomposition differently. Funct Ecol.

[117] Shah F, Nicolás C, Bentzer J, Ellström M, Smits M, Rineau F (2016). Ectomycorrhizal fungi decompose soil organic matter using oxidative mechanisms adapted from saprotrophic ancestors. New Phytol.

[118] Ottosson E, Kubartová A, Edman M, Jönsson M, Lindhe A, Stenlid J (2015). Diverse ecological roles within fungal communities in decomposing logs of *Picea abies*. FEMS Microbiol Ecol.

[119] Brabcová V, Nováková M, Davidová A, Baldrian P (2016). Dead fungal mycelium in forest soil represents a decomposition hotspot and a habitat for a specific microbial community. New Phytol.

[120] Eichorst SA, Kuske CR (2012). Identification of cellulose-responsive bacterial and fungal communities in geographically and edaphically different soils by using stable isotope probing. Appl Environ Microbiol.

[121] DeAngelis KM, Brodie EL, DeSantis TZ, Andersen GL, Lindow SE, Firestone MK (2008). Selective progressive response of soil microbial community to wild oat roots. ISME J.

[122] Lladó S, López-Mondéjar R, Baldrian P (2018). Drivers of microbial community structure in forest soils. Appl Microbiol Biotechnol.

[123] Peura S, Eiler A, Bertilsson S, Nykänen H, Tiirola M, Jones RI (2012). Distinct and diverse anaerobic bacterial communities in boreal lakes dominated by candidate division OD1. ISME J.

[124] Nelson WC, Stegen JC (2015). The reduced genomes of Parcubacteria (OD1) contain signatures of a symbiotic lifestyle. Front Microbiol.

[125] Castelle CJ, Brown CT, Thomas BC, Williams KH, Banfield JF (2017). Unusual respiratory capacity and nitrogen metabolism in a Parcubacterium (OD1) of the Candidate Phyla Radiation. Sci Rep.

[126] Lladó S, López-Mondéjar R, Baldrian P (2017). Forest soil bacteria: diversity, involvement in ecosystem processes, and response to global change. Microbiol Mol Biol Rev.

[127] Arnstadt T, Hoppe B, Kahl T, Kellner H, Krüger D, Bauhus J (2016). Dynamics of fungal community composition, decomposition and resulting deadwood properties in logs of *Fagus sylvatica*, *Picea abies* and *Pinus sylvestris*. Forest Ecol Manag.

[128] Nuccio EE, Hodge A, Pett-Ridge J, Herman DJ, Weber PK, Firestone MK (2013). An arbuscular mycorrhizal fungus significantly modifies the soil bacterial community and nitrogen cycling during litter decomposition. Environ Microbiol.

[129] De Boer W, Folman LB, Summerbell RC, Boddy L (2005). Living in a fungal world: impact of fungi on soil bacterial niche development. FEMS Microbiol Rev.

[130] Tian J-H, Pourcher A-M, Bouchez T, Gelhaye E, Peu P (2014). Occurrence of lignin degradation genotypes and phenotypes among prokaryotes. Appl Microbiol Biotechnol.

[131] Masai E, Katayama Y, Nishikawa S, Fukuda M (1999). Characterization of *Sphingomonas paucimobilis* SYK-6 genes involved in degradation of lignin-related compounds. J Ind Microbiol Biotechnol.

[132] Bandounas L, Wierckx NJ, de Winde JH, Ruijssenaars HJ (2011). Isolation and characterization of novel bacterial strains exhibiting ligninolytic potential. BMC Biotechnol.

[133] Valášková V, De Boer W, Gunnewiek PJK, Pospíšek M, Baldrian P (2009). Phylogenetic composition and properties of bacteria coexisting with the fungus *Hypholoma fasciculare* in decaying wood. ISME J.

[134] Rousk J, Brookes PC, Bååth E (2009). Contrasting soil pH effects on fungal and bacterial growth suggest functional redundancy in carbon mineralization. Appl Environ Microbiol.

[135] Kyaschenko J, Clemmensen KE, Hagenbo A, Karltun E, Lindahl BD (2017). Shift in fungal communities and associated enzyme activities along an age gradient of managed *Pinus sylvestris* stands. ISME J.

