# Supplementary material for: Microbial succession on decomposing root litter in a drought-prone Scots pine forest
Source: ISME J. 2019 May 23;13(9):2346–62. doi: 10.1038/s41396-019-0436-6 (PMC6776048; doi:10.1038/s41396-019-0436-6)
Supplement: Supplementary file 1 — Supplementary materials [file 41396_2019_436_MOESM1_ESM.docx]

# Supplementary materials

Supplemental Table S1: List of monophenols with abbreviations and amounts needed for quantification on GC-FID by external standard mix and internal standard phenyl acetic acid (PAA).

| **Standard Mix** | **Abbreviation** | **Amount** |
| --- | --- | --- |
| p-hydroxybenzaldehyde | PAL | 3 mg |
| p-hydroxyacetophenon | PON | 3 mg |
| vanillin | VAL | 150 mg |
| ethylvanillin | EVAL | 3 mg |
| p-hydroxy benzoic acid | PAD | 20 mg |
| acetovanillon | VON | 20 mg |
| syringaldehyde | SAL | 3 mg |
| vanillic acid | VAD | 20 mg |
| acetosyringone | SON | 3 mg |
| syringic acid | SAD | 3 mg |
| p-coumaric acid | CAD | 3 mg |
| ferulic acid | FAD | 20 mg |
|  |  |  |
| **PAA Standard** | **Abbreviation** | **Amount** |
| phenyl acetic acid | PAA | 3 mg |


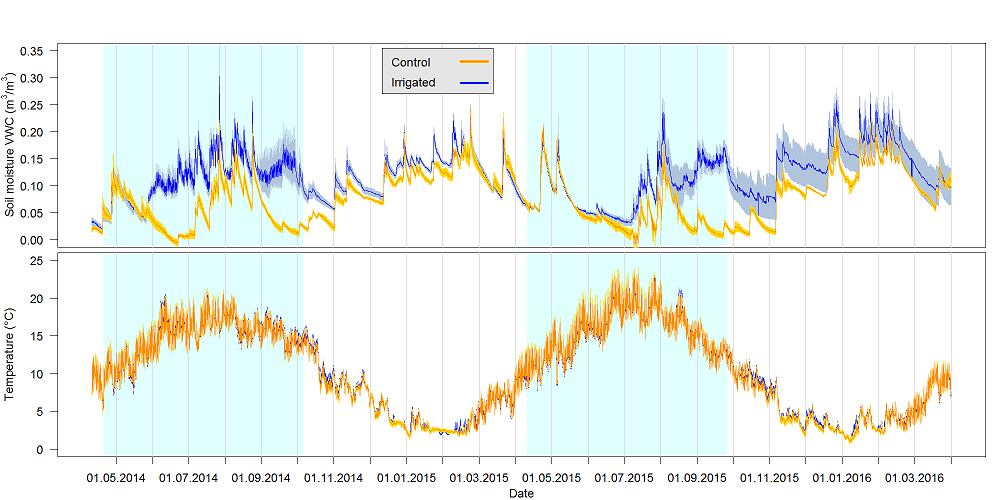


Supplementary Figure S1: Soil volumetric water content and temperature development over the study period at 5 cm soil depth. The hourly mean is plotted with the standard error (shaded areas) for the irrigated plots (blue lines) and the control plots (orange lines). Light blue boxes mark irrigation periods.

Supplementary Figure S2: Bacterial (A) and fungal (B) abundance in decomposing fine roots for the six different time-points and the two different treatments (control and irrigated). Error bars represent standard errors (n=4).

Supplementary Figure S3: Relative change in abundance over the two-year study time of Ascomycete (A) and Basidiomycete (B) abundance on decomposing fine roots. Boxplot with thick black line showing the data median, box is equal to the 50%-quantile, and the whiskers are 1.5 times the interquantile range.
